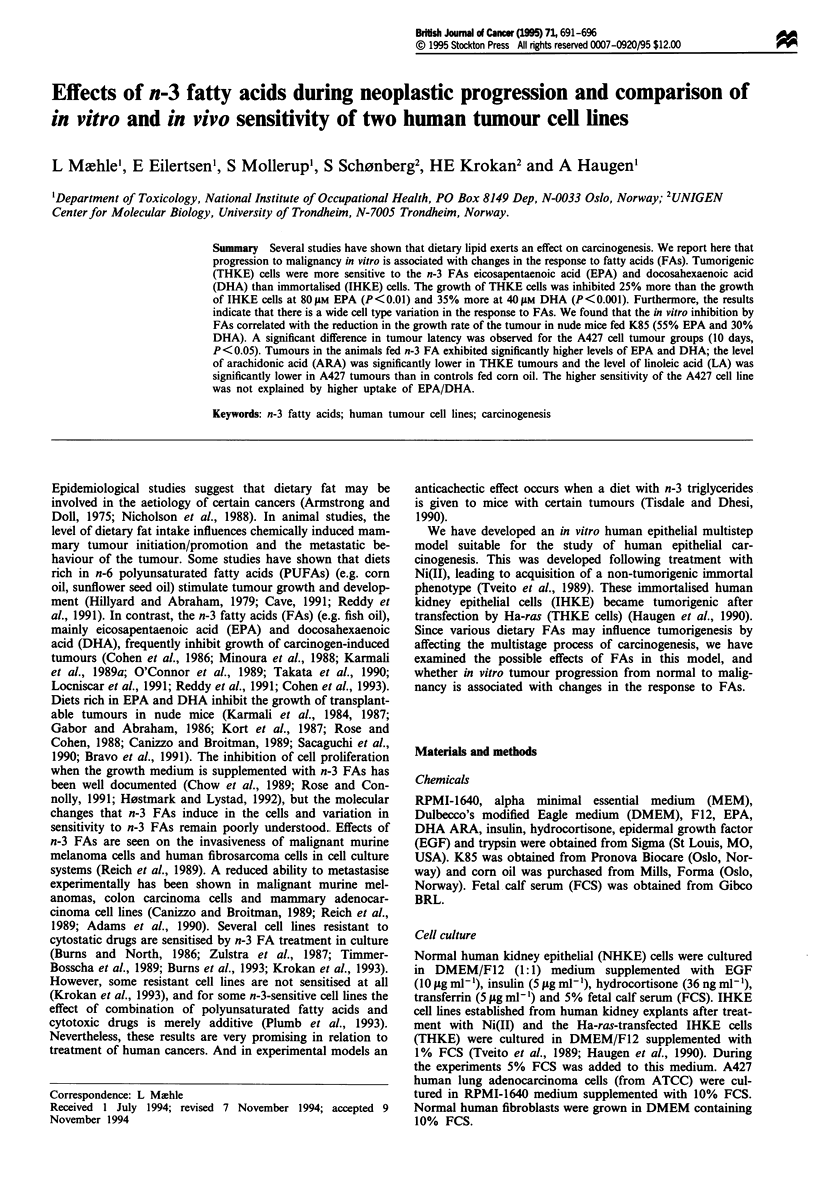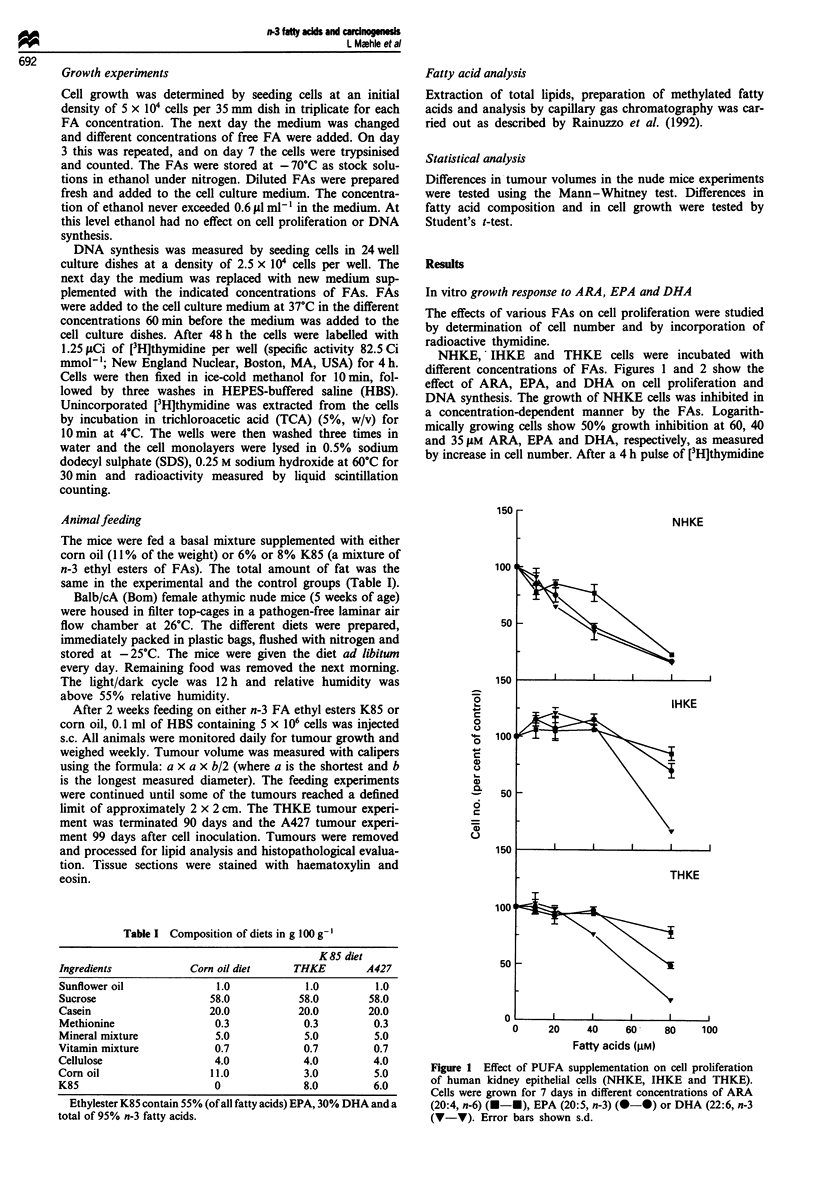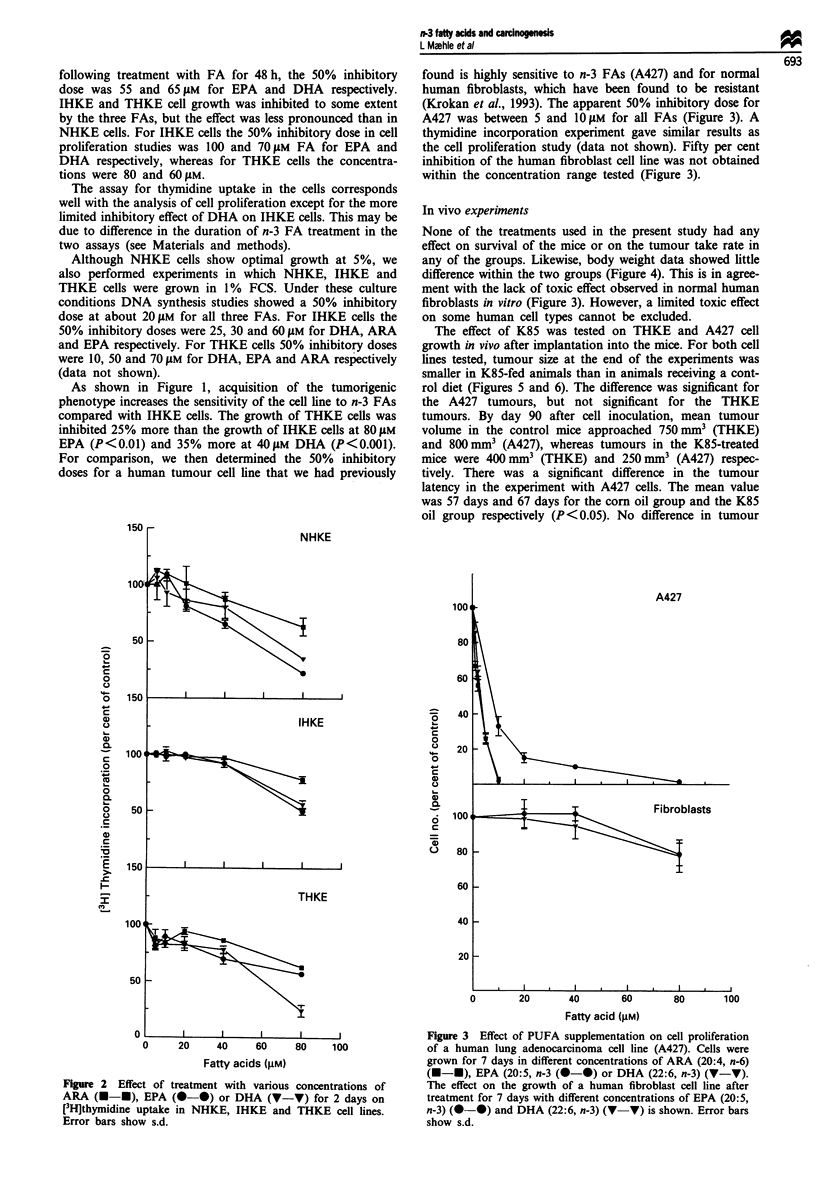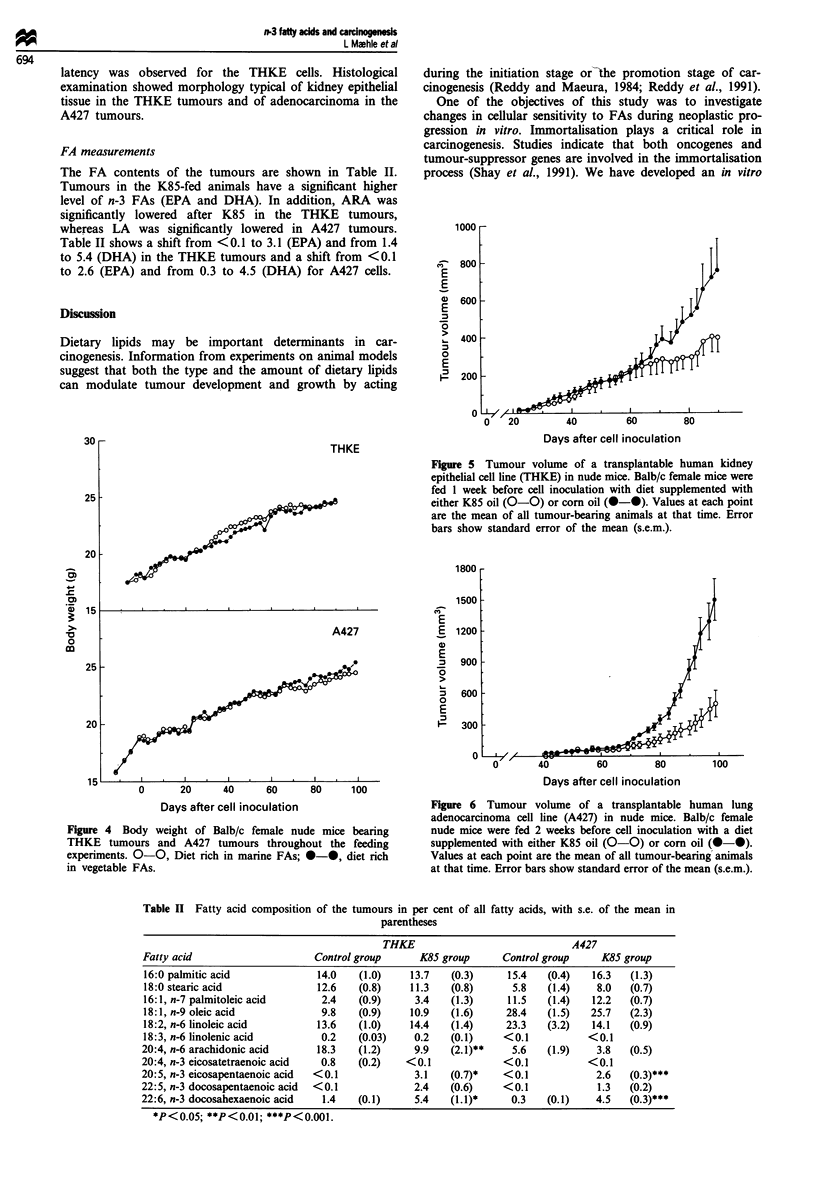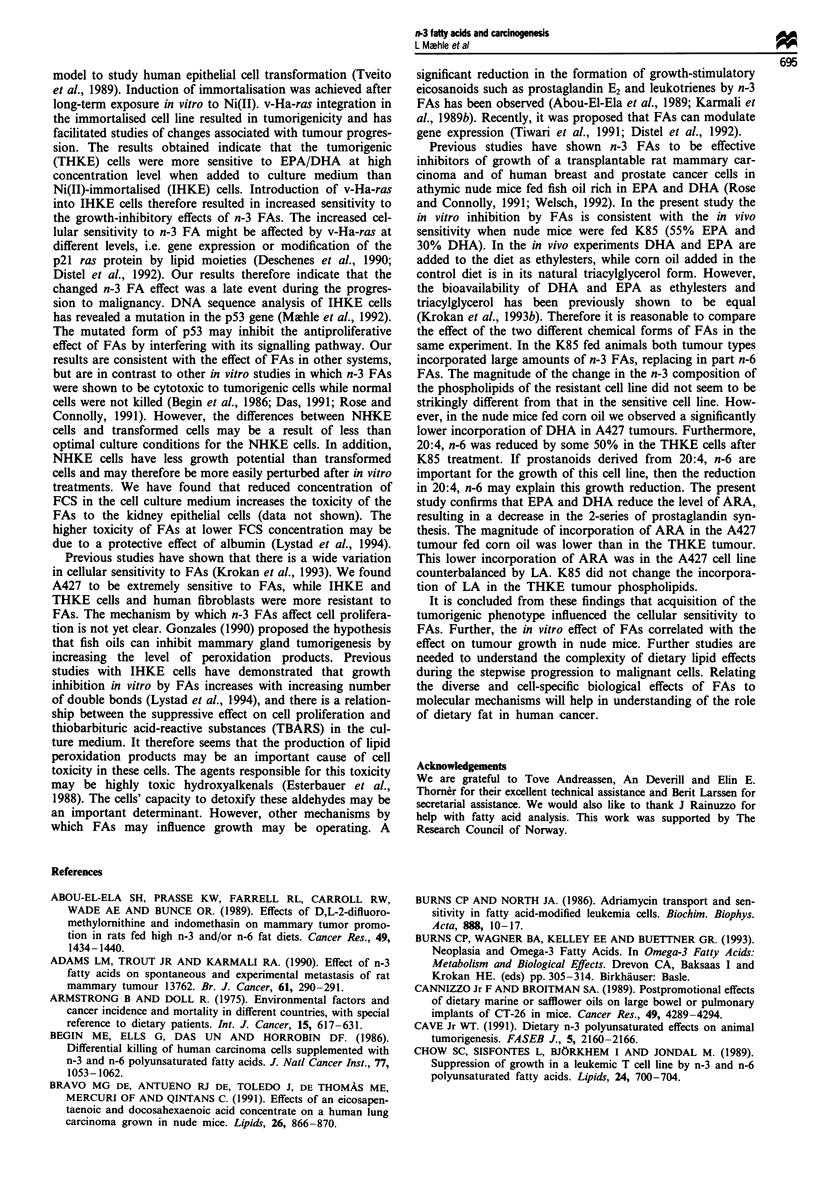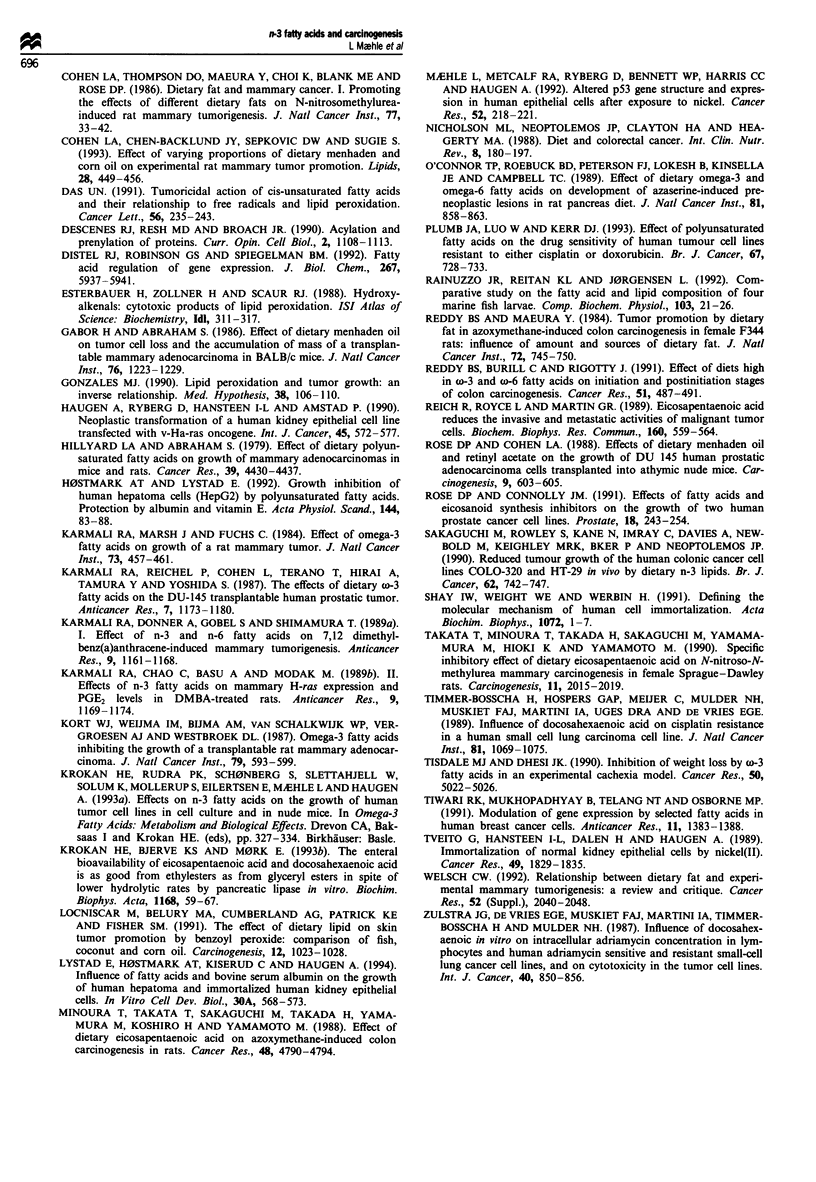# Effects of n-3 fatty acids during neoplastic progression and comparison of in vitro and in vivo sensitivity of two human tumour cell lines.

**DOI:** 10.1038/bjc.1995.136

**Published:** 1995-04

**Authors:** L. Maehle, E. Eilertsen, S. Mollerup, S. Schønberg, H. E. Krokan, A. Haugen

**Affiliations:** Department of Toxicology, National Institute of Occupational Health, Oslo, Norway.

## Abstract

Several studies have shown that dietary lipid exerts an effect on carcinogenesis. We report here that progression to malignancy in vitro is associated with changes in the response to fatty acids (FAs). Tumorigenic (THKE) cells were more sensitive to the n-3 FAs eicosapentaenoic acid (EPA) and docosahexaenoic acid (DHA) than immortalised (IHKE) cells. The growth of THKE cells was inhibited 25% more than the growth of IHKE cells at 80 microM EPA (P < 0.01) and 35% more at 40 microM DHA (P < 0.001). Furthermore, the results indicate that there is a wide cell type variation in the response to FAs. We found that the in vitro inhibition by FAs correlated with the reduction in the growth rate of the tumour in nude mice fed K85 (55% EPA and 30% DHA). A significant difference in tumour latency was observed for the A427 cell tumour groups (10 days, P < 0.05). Tumours in the animals fed n-3 FA exhibited significantly higher levels of EPA and DHA; the level of arachidonic acid (ARA) was significantly lower in THKE tumours and the level of linoleic acid (LA) was significantly lower in A427 tumours than in controls fed corn oil. The higher sensitivity of the A427 cell line was not explained by higher uptake of EPA/DHA.


British Journal of Cancer (1995) 71, 691-696
© 1995 Stockton Press All rights reserved 0007-0920/95 $12.00

### Effects of *n*-3 fatty acids during neoplastic progression and comparison of *in vitro* and *in vivo* sensitivity of two human tumour cell lines

L Mæhle[1], E Eilertsen[1], S Mollerup[1], S Schønberg[2], HE Krokan[2] and A Haugen[1]

[1]*Department of Toxicology, National Institute of Occupational Health, PO Box 8149 Dep, N-0033 Oslo, Norway;* [2]*UNIGEN Center for Molecular Biology, University of Trondheim, N-7005 Trondheim, Norway.*

**Summary** Several studies have shown that dietary lipid exerts an effect on carcinogenesis. We report here that progression to malignancy *in vitro* is associated with changes in the response to fatty acids (FAs). Tumorigenic (THKE) cells were more sensitive to the *n*-3 FAs eicosapentaenoic acid (EPA) and docosahexaenoic acid (DHA) than immortalised (IHKE) cells. The growth of THKE cells was inhibited 25% more than the growth of IHKE cells at 80 μM EPA ($P<0.01$) and 35% more at 40 μM DHA ($P<0.001$). Furthermore, the results indicate that there is a wide cell type variation in the response to FAs. We found that the *in vitro* inhibition by FAs correlated with the reduction in the growth rate of the tumour in nude mice fed K85 (55% EPA and 30% DHA). A significant difference in tumour latency was observed for the A427 cell tumour groups (10 days, $P<0.05$). Tumours in the animals fed *n*-3 FA exhibited significantly higher levels of EPA and DHA; the level of arachidonic acid (ARA) was significantly lower in THKE tumours and the level of linoleic acid (LA) was significantly lower in A427 tumours than in controls fed corn oil. The higher sensitivity of the A427 cell line was not explained by higher uptake of EPA/DHA.

**Keywords:** *n*-3 fatty acids; human tumour cell lines; carcinogenesis

Epidemiological studies suggest that dietary fat may be involved in the aetiology of certain cancers (Armstrong and Doll, 1975; Nicholson *et al.*, 1988). In animal studies, the level of dietary fat intake influences chemically induced mammary tumour initiation/promotion and the metastatic behaviour of the tumour. Some studies have shown that diets rich in *n*-6 polyunsaturated fatty acids (PUFAs) (e.g. corn oil, sunflower seed oil) stimulate tumour growth and development (Hillyard and Abraham, 1979; Cave, 1991; Reddy *et al.*, 1991). In contrast, the *n*-3 fatty acids (FAs) (e.g. fish oil), mainly eicosapentaenoic acid (EPA) and docosahexaenoic acid (DHA), frequently inhibit growth of carcinogen-induced tumours (Cohen *et al.*, 1986; Minoura *et al.*, 1988; Karmali *et al.*, 1989*a*; O'Connor *et al.*, 1989; Takata *et al.*, 1990; Locniscar *et al.*, 1991; Reddy *et al.*, 1991; Cohen *et al.*, 1993). Diets rich in EPA and DHA inhibit the growth of transplantable tumours in nude mice (Karmali *et al.*, 1984, 1987; Gabor and Abraham, 1986; Kort *et al.*, 1987; Rose and Cohen, 1988; Canizzo and Broitman, 1989; Sacaguchi *et al.*, 1990; Bravo *et al.*, 1991). The inhibition of cell proliferation when the growth medium is supplemented with *n*-3 FAs has been well documented (Chow *et al.*, 1989; Rose and Connolly, 1991; Høstmark and Lystad, 1992), but the molecular changes that *n*-3 FAs induce in the cells and variation in sensitivity to *n*-3 FAs remain poorly understood. Effects of *n*-3 FAs are seen on the invasiveness of malignant murine melanoma cells and human fibrosarcoma cells in cell culture systems (Reich *et al.*, 1989). A reduced ability to metastasise experimentally has been shown in malignant murine melanomas, colon carcinoma cells and mammary adenocarcinoma cell lines (Canizzo and Broitman, 1989; Reich *et al.*, 1989; Adams *et al.*, 1990). Several cell lines resistant to cytostatic drugs are sensitised by *n*-3 FA treatment in culture (Burns and North, 1986; Zulstra *et al.*, 1987; Timmer-Bosscha *et al.*, 1989; Burns *et al.*, 1993; Krokan *et al.*, 1993). However, some resistant cell lines are not sensitised at all (Krokan *et al.*, 1993), and for some *n*-3-sensitive cell lines the effect of combination of polyunsaturated fatty acids and cytotoxic drugs is merely additive (Plumb *et al.*, 1993). Nevertheless, these results are very promising in relation to treatment of human cancers. And in experimental models an anticachectic effect occurs when a diet with *n*-3 triglycerides is given to mice with certain tumours (Tisdale and Dhesi, 1990).

We have developed an *in vitro* human epithelial multistep model suitable for the study of human epithelial carcinogenesis. This was developed following treatment with Ni(II), leading to acquisition of a non-tumorigenic immortal phenotype (Tveito *et al.*, 1989). These immortalised human kidney epithelial cells (IHKE) became tumorigenic after transfection by Ha-*ras* (THKE cells) (Haugen *et al.*, 1990). Since various dietary FAs may influence tumorigenesis by affecting the multistage process of carcinogenesis, we have examined the possible effects of FAs in this model, and whether *in vitro* tumour progression from normal to malignancy is associated with changes in the response to FAs.

Correspondence: L Mæhle
Received 1 July 1994; revised 7 November 1994; accepted 9 November 1994

### Materials and methods

### *Chemicals*

RPMI-1640, alpha minimal essential medium (MEM), Dulbecco's modified Eagle medium (DMEM), F12, EPA, DHA ARA, insulin, hydrocortisone, epidermal growth factor (EGF) and trypsin were obtained from Sigma (St Louis, MO, USA). K85 was obtained from Pronova Biocare (Oslo, Norway) and corn oil was purchased from Mills, Forma (Oslo, Norway). Fetal calf serum (FCS) was obtained from Gibco BRL.

### *Cell culture*

Normal human kidney epithelial (NHKE) cells were cultured in DMEM/F12 (1:1) medium supplemented with EGF (10 μg $ml^{-1}$), insulin (5 μg $ml^{-1}$), hydrocortisone (36 ng $ml^{-1}$), transferrin (5 μg $ml^{-1}$) and 5% fetal calf serum (FCS). IHKE cell lines established from human kidney explants after treatment with Ni(II) and the Ha-*ras*-transfected IHKE cells (THKE) were cultured in DMEM/F12 supplemented with 1% FCS (Tveito *et al.*, 1989; Haugen *et al.*, 1990). During the experiments 5% FCS was added to this medium. A427 human lung adenocarcinoma cells (from ATCC) were cultured in RPMI-1640 medium supplemented with 10% FCS. Normal human fibroblasts were grown in DMEM containing 10% FCS.

*Growth experiments*

Cell growth was determined by seeding cells at an initial density of $5 \times 10^4$ cells per 35 mm dish in triplicate for each FA concentration. The next day the medium was changed and different concentrations of free FA were added. On day 3 this was repeated, and on day 7 the cells were trypsinised and counted. The FAs were stored at −70°C as stock solutions in ethanol under nitrogen. Diluted FAs were prepared fresh and added to the cell culture medium. The concentration of ethanol never exceeded 0.6 $\mu l\ ml^{-1}$ in the medium. At this level ethanol had no effect on cell proliferation or DNA synthesis.

DNA synthesis was measured by seeding cells in 24 well culture dishes at a density of $2.5 \times 10^4$ cells per well. The next day the medium was replaced with new medium supplemented with the indicated concentrations of FAs. FAs were added to the cell culture medium at 37°C in the different concentrations 60 min before the medium was added to the cell culture dishes. After 48 h the cells were labelled with 1.25 μCi of [$^3H$]thymidine per well (specific activity 82.5 Ci $mmol^{-1}$; New England Nuclear, Boston, MA, USA) for 4 h. Cells were then fixed in ice-cold methanol for 10 min, followed by three washes in HEPES-buffered saline (HBS). Unincorporated [$^3H$]thymidine was extracted from the cells by incubation in trichloroacetic acid (TCA) (5%, w/v) for 10 min at 4°C. The wells were then washed three times in water and the cell monolayers were lysed in 0.5% sodium dodecyl sulphate (SDS), 0.25 M sodium hydroxide at 60°C for 30 min and radioactivity measured by liquid scintillation counting.

*Animal feeding*

The mice were fed a basal mixture supplemented with either corn oil (11% of the weight) or 6% or 8% K85 (a mixture of *n*-3 ethyl esters of FAs). The total amount of fat was the same in the experimental and the control groups (Table I).

Balb/cA (Bom) female athymic nude mice (5 weeks of age) were housed in filter top-cages in a pathogen-free laminar air flow chamber at 26°C. The different diets were prepared, immediately packed in plastic bags, flushed with nitrogen and stored at −25°C. The mice were given the diet *ad libitum* every day. Remaining food was removed the next morning. The light/dark cycle was 12 h and relative humidity was above 55% relative humidity.

After 2 weeks feeding on either *n*-3 FA ethyl esters K85 or corn oil, 0.1 ml of HBS containing $5 \times 10^6$ cells was injected s.c. All animals were monitored daily for tumour growth and weighed weekly. Tumour volume was measured with calipers using the formula: $a \times a \times b/2$ (where $a$ is the shortest and $b$ is the longest measured diameter). The feeding experiments were continued until some of the tumours reached a defined limit of approximately 2 × 2 cm. The THKE tumour experiment was terminated 90 days and the A427 tumour experiment 99 days after cell inoculation. Tumours were removed and processed for lipid analysis and histopathological evaluation. Tissue sections were stained with haematoxylin and eosin.

**Table I** Composition of diets in g 100 $g^{-1}$

| Ingredients | Corn oil diet | K85 diet THKE | K85 diet A427 |
|---|---|---|---|
| Sunflower oil | 1.0 | 1.0 | 1.0 |
| Sucrose | 58.0 | 58.0 | 58.0 |
| Casein | 20.0 | 20.0 | 20.0 |
| Methionine | 0.3 | 0.3 | 0.3 |
| Mineral mixture | 5.0 | 5.0 | 5.0 |
| Vitamin mixture | 0.7 | 0.7 | 0.7 |
| Cellulose | 4.0 | 4.0 | 4.0 |
| Corn oil | 11.0 | 3.0 | 5.0 |
| K85 | 0 | 8.0 | 6.0 |

Ethylester K85 contain 55% (of all fatty acids) EPA, 30% DHA and a total of 95% *n*-3 fatty acids.

*Fatty acid analysis*

Extraction of total lipids, preparation of methylated fatty acids and analysis by capillary gas chromatography was carried out as described by Rainuzzo *et al.* (1992).

*Statistical analysis*

Differences in tumour volumes in the nude mice experiments were tested using the Mann–Whitney test. Differences in fatty acid composition and in cell growth were tested by Student's *t*-test.

### Results

*In vitro growth response to ARA, EPA and DHA*

The effects of various FAs on cell proliferation were studied by determination of cell number and by incorporation of radioactive thymidine.

NHKE, IHKE and THKE cells were incubated with different concentrations of FAs. Figures 1 and 2 show the effect of ARA, EPA, and DHA on cell proliferation and DNA synthesis. The growth of NHKE cells was inhibited in a concentration-dependent manner by the FAs. Logarithmically growing cells show 50% growth inhibition at 60, 40 and 35 μM ARA, EPA and DHA, respectively, as measured by increase in cell number. After a 4 h pulse of [$^3H$]thymidine

**Figure 1** Effect of PUFA supplementation on cell proliferation of human kidney epithelial cells (NHKE, IHKE and THKE). Cells were grown for 7 days in different concentrations of ARA (20:4, *n*-6) (■—■), EPA (20:5, *n*-3) (●—●) or DHA (22:6, *n*-3) (▼—▼). Error bars shown s.d.

following treatment with FA for 48 h, the 50% inhibitory dose was 55 and 65 µM for EPA and DHA respectively. IHKE and THKE cell growth was inhibited to some extent by the three FAs, but the effect was less pronounced than in NHKE cells. For IHKE cells the 50% inhibitory dose in cell proliferation studies was 100 and 70 µM FA for EPA and DHA respectively, whereas for THKE cells the concentrations were 80 and 60 µM.

The assay for thymidine uptake in the cells corresponds well with the analysis of cell proliferation except for the more limited inhibitory effect of DHA on IHKE cells. This may be due to difference in the duration of n-3 FA treatment in the two assays (see Materials and methods).

Although NHKE cells show optimal growth at 5%, we also performed experiments in which NHKE, IHKE and THKE cells were grown in 1% FCS. Under these culture conditions DNA synthesis studies showed a 50% inhibitory dose at about 20 µM for all three FAs. For IHKE cells the 50% inhibitory doses were 25, 30 and 60 µM for DHA, ARA and EPA respectively. For THKE cells 50% inhibitory doses were 10, 50 and 70 µM for DHA, EPA and ARA respectively (data not shown).

As shown in Figure 1, acquisition of the tumorigenic phenotype increases the sensitivity of the cell line to n-3 FAs compared with IHKE cells. The growth of THKE cells was inhibited 25% more than the growth of IHKE cells at 80 µM EPA ($P<0.01$) and 35% more at 40 µM DHA ($P<0.001$). For comparison, we then determined the 50% inhibitory doses for a human tumour cell line that we had previously found is highly sensitive to n-3 FAs (A427) and for normal human fibroblasts, which have been found to be resistant (Krokan *et al.*, 1993). The apparent 50% inhibitory dose for A427 was between 5 and 10 µM for all FAs (Figure 3). A thymidine incorporation experiment gave similar results as the cell proliferation study (data not shown). Fifty per cent inhibition of the human fibroblast cell line was not obtained within the concentration range tested (Figure 3).

### In vivo *experiments*

None of the treatments used in the present study had any effect on survival of the mice or on the tumour take rate in any of the groups. Likewise, body weight data showed little difference within the two groups (Figure 4). This is in agreement with the lack of toxic effect observed in normal human fibroblasts *in vitro* (Figure 3). However, a limited toxic effect on some human cell types cannot be excluded.

The effect of K85 was tested on THKE and A427 cell growth *in vivo* after implantation into the mice. For both cell lines tested, tumour size at the end of the experiments was smaller in K85-fed animals than in animals receiving a control diet (Figures 5 and 6). The difference was significant for the A427 tumours, but not significant for the THKE tumours. By day 90 after cell inoculation, mean tumour volume in the control mice approached 750 $mm^3$ (THKE) and 800 $mm^3$ (A427), whereas tumours in the K85-treated mice were 400 $mm^3$ (THKE) and 250 $mm^3$ (A427) respectively. There was a significant difference in the tumour latency in the experiment with A427 cells. The mean value was 57 days and 67 days for the corn oil group and the K85 oil group respectively ($P<0.05$). No difference in tumour

**Figure 2** Effect of treatment with various concentrations of ARA (■—■), EPA (●—●) or DHA (▼—▼) for 2 days on [$^3$H]thymidine uptake in NHKE, IHKE and THKE cell lines. Error bars show s.d.

**Figure 3** Effect of PUFA supplementation on cell proliferation of a human lung adenocarcinoma cell line (A427). Cells were grown for 7 days in different concentrations of ARA (20:4, n-6) (■—■), EPA (20:5, n-3 (●—●) or DHA (22:6, n-3) (▼—▼). The effect on the growth of a human fibroblast cell line after treatment for 7 days with different concentrations of EPA (20:5, n-3) (●—●) and DHA (22:6, n-3) (▼—▼) is shown. Error bars show s.d.

latency was observed for the THKE cells. Histological examination showed morphology typical of kidney epithelial tissue in the THKE tumours and of adenocarcinoma in the A427 tumours.

### *FA measurements*

The FA contents of the tumours are shown in Table II. Tumours in the K85-fed animals have a significant higher level of *n*-3 FAs (EPA and DHA). In addition, ARA was significantly lowered after K85 in the THKE tumours, whereas LA was significantly lowered in A427 tumours. Table II shows a shift from <0.1 to 3.1 (EPA) and from 1.4 to 5.4 (DHA) in the THKE tumours and a shift from <0.1 to 2.6 (EPA) and from 0.3 to 4.5 (DHA) for A427 cells.

### Discussion

Dietary lipids may be important determinants in carcinogenesis. Information from experiments on animal models suggest that both the type and the amount of dietary lipids can modulate tumour development and growth by acting during the initiation stage or the promotion stage of carcinogenesis (Reddy and Maeura, 1984; Reddy *et al.*, 1991).

One of the objectives of this study was to investigate changes in cellular sensitivity to FAs during neoplastic progression *in vitro*. Immortalisation plays a critical role in carcinogenesis. Studies indicate that both oncogenes and tumour-suppressor genes are involved in the immortalisation process (Shay *et al.*, 1991). We have developed an *in vitro*

**Figure 4** Body weight of Balb/c female nude mice bearing THKE tumours and A427 tumours throughout the feeding experiments. O—O, Diet rich in marine FAs; ●—●, diet rich in vegetable FAs.

**Figure 5** Tumour volume of a transplantable human kidney epithelial cell line (THKE) in nude mice. Balb/c female mice were fed 1 week before cell inoculation with diet supplemented with either K85 oil (O—O) or corn oil (●—●). Values at each point are the mean of all tumour-bearing animals at that time. Error bars show standard error of the mean (s.e.m.).

**Figure 6** Tumour volume of a transplantable human lung adenocarcinoma cell line (A427) in nude mice. Balb/c female nude mice were fed 2 weeks before cell inoculation with a diet supplemented with either K85 oil (O—O) or corn oil (●—●). Values at each point are the mean of all tumour-bearing animals at that time. Error bars show standard error of the mean (s.e.m.).

**Table II** Fatty acid composition of the tumours in per cent of all fatty acids, with s.e. of the mean in parentheses

| | *THKE* | | | | *A427* | | | |
|---|---|---|---|---|---|---|---|---|
| *Fatty acid* | *Control group* | | *K85 group* | | *Control group* | | *K85 group* | |
| 16:0 palmitic acid | 14.0 | (1.0) | 13.7 | (0.3) | 15.4 | (0.4) | 16.3 | (1.3) |
| 18:0 stearic acid | 12.6 | (0.8) | 11.3 | (0.8) | 5.8 | (1.4) | 8.0 | (0.7) |
| 16:1, *n*-7 palmitoleic acid | 2.4 | (0.9) | 3.4 | (1.3) | 11.5 | (1.4) | 12.2 | (0.7) |
| 18:1, *n*-9 oleic acid | 9.8 | (0.9) | 10.9 | (1.6) | 28.4 | (1.5) | 25.7 | (2.3) |
| 18:2, *n*-6 linoleic acid | 13.6 | (1.0) | 14.4 | (1.4) | 23.3 | (3.2) | 14.1 | (0.9) |
| 18:3, *n*-6 linolenic acid | 0.2 | (0.03) | 0.2 | (0.1) | <0.1 | | <0.1 | |
| 20:4, *n*-6 arachidonic acid | 18.3 | (1.2) | 9.9 | (2.1)** | 5.6 | (1.9) | 3.8 | (0.5) |
| 20:4, *n*-3 eicosatetraenoic acid | 0.8 | (0.2) | <0.1 | | <0.1 | | <0.1 | |
| 20:5, *n*-3 eicosapentaenoic acid | <0.1 | | 3.1 | (0.7)* | <0.1 | | 2.6 | (0.3)*** |
| 22:5, *n*-3 docosapentaenoic acid | <0.1 | | 2.4 | (0.6) | <0.1 | | 1.3 | (0.2) |
| 22:6, *n*-3 docosahexaenoic acid | 1.4 | (0.1) | 5.4 | (1.1)* | 0.3 | (0.1) | 4.5 | (0.3)*** |

*$P<0.05$; **$P<0.01$; ***$P<0.001$.

model to study human epithelial cell transformation (Tveito *et al.*, 1989). Induction of immortalisation was achieved after long-term exposure *in vitro* to Ni(II). v-Ha-*ras* integration in the immortalised cell line resulted in tumorigenicity and has facilitated studies of changes associated with tumour progression. The results obtained indicate that the tumorigenic (THKE) cells were more sensitive to EPA/DHA at high concentration level when added to culture medium than Ni(II)-immortalised (IHKE) cells. Introduction of v-Ha-*ras* into IHKE cells therefore resulted in increased sensitivity to the growth-inhibitory effects of *n*-3 FAs. The increased cellular sensitivity to *n*-3 FA might be affected by v-Ha-*ras* at different levels, i.e. gene expression or modification of the p21 *ras* protein by lipid moieties (Deschenes *et al.*, 1990; Distel *et al.*, 1992). Our results therefore indicate that the changed *n*-3 FA effect was a late event during the progression to malignancy. DNA sequence analysis of IHKE cells has revealed a mutation in the p53 gene (Mæhle *et al.*, 1992). The mutated form of p53 may inhibit the antiproliferative effect of FAs by interfering with its signalling pathway. Our results are consistent with the effect of FAs in other systems, but are in contrast to other *in vitro* studies in which *n*-3 FAs were shown to be cytotoxic to tumorigenic cells while normal cells were not killed (Begin *et al.*, 1986; Das, 1991; Rose and Connolly, 1991). However, the differences between NHKE cells and transformed cells may be a result of less than optimal culture conditions for the NHKE cells. In addition, NHKE cells have less growth potential than transformed cells and may therefore be more easily perturbed after *in vitro* treatments. We have found that reduced concentration of FCS in the cell culture medium increases the toxicity of the FAs to the kidney epithelial cells (data not shown). The higher toxicity of FAs at lower FCS concentration may be due to a protective effect of albumin (Lystad *et al.*, 1994).

Previous studies have shown that there is a wide variation in cellular sensitivity to FAs (Krokan *et al.*, 1993). We found A427 to be extremely sensitive to FAs, while IHKE and THKE cells and human fibroblasts were more resistant to FAs. The mechanism by which *n*-3 FAs affect cell proliferation is not yet clear. Gonzales (1990) proposed the hypothesis that fish oils can inhibit mammary gland tumorigenesis by increasing the level of peroxidation products. Previous studies with IHKE cells have demonstrated that growth inhibition *in vitro* by FAs increases with increasing number of double bonds (Lystad *et al.*, 1994), and there is a relationship between the suppressive effect on cell proliferation and thiobarbituric acid-reactive substances (TBARS) in the culture medium. It therefore seems that the production of lipid peroxidation products may be an important cause of cell toxicity in these cells. The agents responsible for this toxicity may be highly toxic hydroxyalkenals (Esterbauer *et al.*, 1988). The cells' capacity to detoxify these aldehydes may be an important determinant. However, other mechanisms by which FAs may influence growth may be operating. A significant reduction in the formation of growth-stimulatory eicosanoids such as prostaglandin $E_2$ and leukotrienes by *n*-3 FAs has been observed (Abou-El-Ela *et al.*, 1989; Karmali *et al.*, 1989*b*). Recently, it was proposed that FAs can modulate gene expression (Tiwari *et al.*, 1991; Distel *et al.*, 1992).

Previous studies have shown *n*-3 FAs to be effective inhibitors of growth of a transplantable rat mammary carcinoma and of human breast and prostate cancer cells in athymic nude mice fed fish oil rich in EPA and DHA (Rose and Connolly, 1991; Welsch, 1992). In the present study the *in vitro* inhibition by FAs is consistent with the *in vivo* sensitivity when nude mice were fed K85 (55% EPA and 30% DHA). In the *in vivo* experiments DHA and EPA are added to the diet as ethylesters, while corn oil added in the control diet is in its natural triacylglycerol form. However, the bioavailability of DHA and EPA as ethylesters and triacylglycerol has been previously shown to be equal (Krokan *et al.*, 1993*b*). Therefore it is reasonable to compare the effect of the two different chemical forms of FAs in the same experiment. In the K85 fed animals both tumour types incorporated large amounts of *n*-3 FAs, replacing in part *n*-6 FAs. The magnitude of the change in the *n*-3 composition of the phospholipids of the resistant cell line did not seem to be strikingly different from that in the sensitive cell line. However, in the nude mice fed corn oil we observed a significantly lower incorporation of DHA in A427 tumours. Furthermore, 20:4, *n*-6 was reduced by some 50% in the THKE cells after K85 treatment. If prostanoids derived from 20:4, *n*-6 are important for the growth of this cell line, then the reduction in 20:4, *n*-6 may explain this growth reduction. The present study confirms that EPA and DHA reduce the level of ARA, resulting in a decrease in the 2-series of prostaglandin synthesis. The magnitude of incorporation of ARA in the A427 tumour fed corn oil was lower than in the THKE tumour. This lower incorporation of ARA was in the A427 cell line counterbalanced by LA. K85 did not change the incorporation of LA in the THKE tumour phospholipids.

It is concluded from these findings that acquisition of the tumorigenic phenotype influenced the cellular sensitivity to FAs. Further, the *in vitro* effect of FAs correlated with the effect on tumour growth in nude mice. Further studies are needed to understand the complexity of dietary lipid effects during the stepwise progression to malignant cells. Relating the diverse and cell-specific biological effects of FAs to molecular mechanisms will help in understanding of the role of dietary fat in human cancer.

**Acknowledgements**
We are grateful to Tove Andreassen, An Deverill and Elin E. Thornèr for their excellent technical assistance and Berit Larssen for secretarial assistance. We would also like to thank J Rainuzzo for help with fatty acid analysis. This work was supported by The Research Council of Norway.

**References**

ABOU-EL-ELA SH, PRASSE KW, FARRELL RL, CARROLL RW, WADE AE AND BUNCE OR. (1989). Effects of D,L-2-difluoromethylornithine and indomethasin on mammary tumor promotion in rats fed high n-3 and/or n-6 fat diets. *Cancer Res.*, **49**, 1434–1440.

ADAMS LM, TROUT JR AND KARMALI RA. (1990). Effect of n-3 fatty acids on spontaneous and experimental metastasis of rat mammary tumour 13762. *Br. J. Cancer*, **61**, 290–291.

ARMSTRONG B AND DOLL R. (1975). Environmental factors and cancer incidence and mortality in different countries, with special reference to dietary patients. *Int. J. Cancer*, **15**, 617–631.

BEGIN ME, ELLS G, DAS UN AND HORROBIN DF. (1986). Differential killing of human carcinoma cells supplemented with n-3 and n-6 polyunsaturated fatty acids. *J. Natl Cancer Inst.*, **77**, 1053–1062.

BRAVO MG DE, ANTUENO RJ DE, TOLEDO J, DE THOMÀS ME, MERCURI OF AND QINTANS C. (1991). Effects of an eicosapentaenoic and docosahexaenoic acid concentrate on a human lung carcinoma grown in nude mice. *Lipids*, **26**, 866–870.

BURNS CP AND NORTH JA. (1986). Adriamycin transport and sensitivity in fatty acid-modified leukemia cells. *Biochim. Biophys. Acta*, **888**, 10–17.

BURNS CP, WAGNER BA, KELLEY EE AND BUETTNER GR. (1993). Neoplasia and Omega-3 Fatty Acids. In *Omega-3 Fatty Acids: Metabolism and Biological Effects*. Drevon CA, Baksaas I and Krokan HE. (eds) pp. 305–314. Birkhäuser: Basle.

CANNIZZO Jr F AND BROITMAN SA. (1989). Postpromotional effects of dietary marine or safflower oils on large bowel or pulmonary implants of CT-26 in mice. *Cancer Res.*, **49**, 4289–4294.

CAVE Jr WT. (1991). Dietary n-3 polyunsaturated effects on animal tumorigenesis. *FASEB J.*, **5**, 2160–2166.

CHOW SC, SISFONTES L, BJÖRKHEM I AND JONDAL M. (1989). Suppression of growth in a leukemic T cell line by n-3 and n-6 polyunsaturated fatty acids. *Lipids*, **24**, 700–704.

COHEN LA, THOMPSON DO, MAEURA Y, CHOI K, BLANK ME AND ROSE DP. (1986). Dietary fat and mammary cancer. I. Promoting the effects of different dietary fats on N-nitrosomethylurea-induced rat mammary tumorigenesis. *J. Natl Cancer Inst.*, **77**, 33–42.

COHEN LA, CHEN-BACKLUND JY, SEPKOVIC DW AND SUGIE S. (1993). Effect of varying proportions of dietary menhaden and corn oil on experimental rat mammary tumor promotion. *Lipids*, **28**, 449–456.

DAS UN. (1991). Tumoricidal action of cis-unsaturated fatty acids and their relationship to free radicals and lipid peroxidation. *Cancer Lett.*, **56**, 235–243.

DESCENES RJ, RESH MD AND BROACH JR. (1990). Acylation and prenylation of proteins. *Curr. Opin. Cell Biol.*, **2**, 1108–1113.

DISTEL RJ, ROBINSON GS AND SPIEGELMAN BM. (1992). Fatty acid regulation of gene expression. *J. Biol. Chem.*, **267**, 5937–5941.

ESTERBAUER H, ZOLLNER H AND SCAUR RJ. (1988). Hydroxy-alkenals: cytotoxic products of lipid peroxidation. *ISI Atlas of Science: Biochemistry*, **Idl**, 311–317.

GABOR H AND ABRAHAM S. (1986). Effect of dietary menhaden oil on tumor cell loss and the accumulation of mass of a transplantable mammary adenocarcinoma in BALB/c mice. *J. Natl Cancer Inst.*, **76**, 1223–1229.

GONZALES MJ. (1990). Lipid peroxidation and tumor growth: an inverse relationship. *Med. Hypothesis*, **38**, 106–110.

HAUGEN A, RYBERG D, HANSTEEN I-L AND AMSTAD P. (1990). Neoplastic transformation of a human kidney epithelial cell line transfected with v-Ha-ras oncogene. *Int. J. Cancer*, **45**, 572–577.

HILLYARD LA AND ABRAHAM S. (1979). Effect of dietary polyunsaturated fatty acids on growth of mammary adenocarcinomas in mice and rats. *Cancer Res.*, **39**, 4430–4437.

HØSTMARK AT AND LYSTAD E. (1992). Growth inhibition of human hepatoma cells (HepG2) by polyunsaturated fatty acids. Protection by albumin and vitamin E. *Acta Physiol. Scand.*, **144**, 83–88.

KARMALI RA, MARSH J AND FUCHS C. (1984). Effect of omega-3 fatty acids on growth of a rat mammary tumor. *J. Natl Cancer Inst.*, **73**, 457–461.

KARMALI RA, REICHEL P, COHEN L, TERANO T, HIRAI A, TAMURA Y AND YOSHIDA S. (1987). The effects of dietary ω-3 fatty acids on the DU-145 transplantable human prostatic tumor. *Anticancer Res.*, **7**, 1173–1180.

KARMALI RA, DONNER A, GOBEL S AND SHIMAMURA T. (1989*a*). I. Effect of n-3 and n-6 fatty acids on 7,12 dimethylbenz(a)anthracene-induced mammary tumorigenesis. *Anticancer Res.*, **9**, 1161–1168.

KARMALI RA, CHAO C, BASU A AND MODAK M. (1989*b*). II. Effects of n-3 fatty acids on mammary H-*ras* expression and $PGE_2$ levels in DMBA-treated rats. *Anticancer Res.*, **9**, 1169–1174.

KORT WJ, WEIJMA IM, BIJMA AM, VAN SCHALKWIJK WP, VERGROESEN AJ AND WESTBROEK DL. (1987). Omega-3 fatty acids inhibiting the growth of a transplantable rat mammary adenocarcinoma. *J. Natl Cancer Inst.*, **79**, 593–599.

KROKAN HE, RUDRA PK, SCHØNBERG S, SLETTAHJELL W, SOLUM K, MOLLERUP S, EILERTSEN E, MÆHLE L AND HAUGEN A. (1993*a*). Effects on n-3 fatty acids on the growth of human tumor cell lines in cell culture and in nude mice. In *Omega-3 Fatty Acids: Metabolism and Biological Effects*. Drevon CA, Baksaas I and Krokan HE. (eds), pp. 327–334. Birkhäuser: Basle.

KROKAN HE, BJERVE KS AND MØRK E. (1993*b*). The enteral bioavailability of eicosapentaenoic acid and docosahexaenoic acid is as good from ethylesters as from glyceryl esters in spite of lower hydrolytic rates by pancreatic lipase *in vitro*. *Biochim. Biophys. Acta*, **1168**, 59–67.

LOCNISCAR M, BELURY MA, CUMBERLAND AG, PATRICK KE AND FISHER SM. (1991). The effect of dietary lipid on skin tumor promotion by benzoyl peroxide: comparison of fish, coconut and corn oil. *Carcinogenesis*, **12**, 1023–1028.

LYSTAD E, HØSTMARK AT, KISERUD C AND HAUGEN A. (1994). Influence of fatty acids and bovine serum albumin on the growth of human hepatoma and immortalized human kidney epithelial cells. *In Vitro Cell Dev. Biol.*, **30A**, 568–573.

MINOURA T, TAKATA T, SAKAGUCHI M, TAKADA H, YAMAMURA M, KOSHIRO H AND YAMAMOTO M. (1988). Effect of dietary eicosapentaenoic acid on azoxymethane-induced colon carcinogenesis in rats. *Cancer Res.*, **48**, 4790–4794.

MÆHLE L, METCALF RA, RYBERG D, BENNETT WP, HARRIS CC AND HAUGEN A. (1992). Altered p53 gene structure and expression in human epithelial cells after exposure to nickel. *Cancer Res.*, **52**, 218–221.

NICHOLSON ML, NEOPTOLEMOS JP, CLAYTON HA AND HEAGERTY MA. (1988). Diet and colorectal cancer. *Int. Clin. Nutr. Rev.*, **8**, 180–197.

O'CONNOR TP, ROEBUCK BD, PETERSON FJ, LOKESH B, KINSELLA JE AND CAMPBELL TC. (1989). Effect of dietary omega-3 and omega-6 fatty acids on development of azaserine-induced preneoplastic lesions in rat pancreas diet. *J. Natl Cancer Inst.*, **81**, 858–863.

PLUMB JA, LUO W AND KERR DJ. (1993). Effect of polyunsaturated fatty acids on the drug sensitivity of human tumour cell lines resistant to either cisplatin or doxorubicin. *Br. J. Cancer*, **67**, 728–733.

RAINUZZO JR, REITAN KL AND JØRGENSEN L. (1992). Comparative study on the fatty acid and lipid composition of four marine fish larvae. *Comp. Biochem. Physiol.*, **103**, 21–26.

REDDY BS AND MAEURA Y. (1984). Tumor promotion by dietary fat in azoxymethane-induced colon carcinogenesis in female F344 rats: influence of amount and sources of dietary fat. *J. Natl Cancer Inst.*, **72**, 745–750.

REDDY BS, BURILL C AND RIGOTTY J. (1991). Effect of diets high in ω-3 and ω-6 fatty acids on initiation and postinitiation stages of colon carcinogenesis. *Cancer Res.*, **51**, 487–491.

REICH R, ROYCE L AND MARTIN GR. (1989). Eicosapentaenoic acid reduces the invasive and metastatic activities of malignant tumor cells. *Biochem. Biophys. Res. Commun.*, **160**, 559–564.

ROSE DP AND COHEN LA. (1988). Effects of dietary menhaden oil and retinyl acetate on the growth of DU 145 human prostatic adenocarcinoma cells transplanted into athymic nude mice. *Carcinogenesis*, **9**, 603–605.

ROSE DP AND CONNOLLY JM. (1991). Effects of fatty acids and eicosanoid synthesis inhibitors on the growth of two human prostate cancer cell lines. *Prostate*, **18**, 243–254.

SAKAGUCHI M, ROWLEY S, KANE N, IMRAY C, DAVIES A, NEWBOLD M, KEIGHLEY MRK, BKER P AND NEOPTOLEMOS JP. (1990). Reduced tumour growth of the human colonic cancer cell lines COLO-320 and HT-29 *in vivo* by dietary n-3 lipids. *Br. J. Cancer*, **62**, 742–747.

SHAY IW, WEIGHT WE AND WERBIN H. (1991). Defining the molecular mechanism of human cell immortalization. *Acta Biochim. Biophys.*, **1072**, 1–7.

TAKATA T, MINOURA T, TAKADA H, SAKAGUCHI M, YAMAMAMURA M, HIOKI K AND YAMAMOTO M. (1990). Specific inhibitory effect of dietary eicosapentaenoic acid on *N*-nitroso-*N*-methylurea mammary carcinogenesis in female Sprague–Dawley rats. *Carcinogenesis*, **11**, 2015–2019.

TIMMER-BOSSCHA H, HOSPERS GAP, MEIJER C, MULDER NH, MUSKIET FAJ, MARTINI IA, UGES DRA AND DE VRIES EGE. (1989). Influence of docosahexaenoic acid on cisplatin resistance in a human small cell lung carcinoma cell line. *J. Natl Cancer Inst.*, **81**, 1069–1075.

TISDALE MJ AND DHESI JK. (1990). Inhibition of weight loss by ω-3 fatty acids in an experimental cachexia model. *Cancer Res.*, **50**, 5022–5026.

TIWARI RK, MUKHOPADHYAY B, TELANG NT AND OSBORNE MP. (1991). Modulation of gene expression by selected fatty acids in human breast cancer cells. *Anticancer Res.*, **11**, 1383–1388.

TVEITO G, HANSTEEN I-L, DALEN H AND HAUGEN A. (1989). Immortalization of normal kidney epithelial cells by nickel(II). *Cancer Res.*, **49**, 1829–1835.

WELSCH CW. (1992). Relationship between dietary fat and experimental mammary tumorigenesis: a review and critique. *Cancer Res.*, **52** (Suppl.), 2040–2048.

ZULSTRA JG, DE VRIES EGE, MUSKIET FAJ, MARTINI IA, TIMMER-BOSSCHA H AND MULDER NH. (1987). Influence of docosahexaenoic *in vitro* on intracellular adriamycin concentration in lymphocytes and human adriamycin sensitive and resistant small-cell lung cancer cell lines, and on cytotoxicity in the tumor cell lines. *Int. J. Cancer*, **40**, 850–856.